# Topics of the Time

**Published:** 1898-01

**Authors:** 


### TOPICS OF THE TIME.

The necessity of drinking water in appropriate quantities is not appreciated by the majority of people. Its importance in the promotion of health and the prevention of disease is not even correctly estimated by some physicians. For this reason, it is well that such avenues as the *Ladies' Home Journal*, with its large circulation, permeating so many thousands of households, should be made available in the dissemination of knowledge on the subject. Mrs. S. T. Rorer, in the December number, discourses upon this most important subject in the following intelligent manner :

> Water is really our only true beverage. Forming, as it does, three-quarters of the weight of the human body, it is of the next importance to the air we breathe. Milk is a typical food, not a beverage, and should never be used as such. It is true that it contains a large amount of water, but only sufficient for its digestion.
>
> In a very short time the non-water-drinker becomes sallow, constipated and uncomfortable. The poison matter that should be dissolved by the free use of water and carried off in the circulation and through the excretory organs is held in the system ; the body loses weight, the skin becomes dry and rough, losing its life and brilliancy. Three-quarters of the weight of the living body should be water. A large quantity of this water is taken in the form of green vegetables and fruits. A healthy person should drink at least a quart and a half of cool (not iced) water in each twenty-four hours—a glass the first thing in the morning and the last thing at night and the remaining quantity after or between meals. Infants frequently suffer more from the lack of cool water than from the lack of food.

---

The paper read by Hon. Wm. P. Letchworth before the national conference of charities and corrections, at Toronto last July, on Dependent children and family homes, contains much to interest those not directly engaged in philanthropic work. Mr. Letchworth's long experience as a state commissioner of charities has shown him the great wrongs which dependent children suffer from being placed in unsuitable homes. These evils arise from indifference before placing them and inattention and neglect afterward. The remedy lies in legislation which will put the placing out of these unfortunate children under the supervision of the state board of charities.

The orphan asylum is no longer regarded as the permanent home for the dependent child, but rather as a temporary refuge and training-school for preparing the child for admittance into a desirable home ; and, further, it has an important work in preventing the breaking up of families, by providing shelter for the children during times of distress and bereavement. Mr. Letchworth notes that Buffalo has a smaller number of children in orphan asylums today, in proportion to its population, than it had twenty years ago.

---

The President in his annual message to the congress paid especial attention to the importance of quarantine reform. The recent yellow fever epidemic at the South has directed public attention again to the inadequacy of the present system of quarantine by the states. It is a generally admitted fact that the epidemic referred to did not receive the skilful handling with reference to its prevention, not to speak of its eradication, that the public is entitled to expect in the present advanced state of the science of public sanitation.

Yellow fever is a disease that is practically unknown in Europe, and it is a disgrace to the United States that the end of the nineteenth century witnesses such an intimate acquaintance with it. We aim our criticisms at no one in particular and without disparagement to the noble efforts made in most regions of the South to stay the recent plague, but only desire to call attention to the general fact that our present methods are inefficient, unscientific, if not disgraceful.

The surgeon-general of the army, Dr. George M. Sternberg, and the secretary of the treasury, Mr. Gage, are at one in recommending a national system of quarantine. The president lends his powerful hand at pulling the oars in that direction, and if we do not speedily extricate ourselves from this dilemma it will not be difficult to demonstrate that it will be entirely the fault of the congress.

The president also adds the recommendation that a systematic investigation of the obscure and hitherto undetermined cause of yellow fever should be made by a commission of four expert bacteriologists appointed by the president from the medical departments of the army and navy, the marine hospital service and civil life. It would cost only a trifling sum to carry out this suggestion, and the results might be of advantage to the whole world.

---

WILLIS S. ANDERSON, of Detroit, has recently published in the *Physician and Surgeon* an article entitled, A study of crime and degenerates from a medical standpoint, in which he deduces strong arguments in favor of heredity as related to the social condition of mankind. In the course of his dissertation he cites the following in support of this theory:

The Jukes family, as studied by Dugdale, is an excellent illustration of the influence of heredity. From the head of the family, Max Jukes, a great drunkard, descended, in seventy-five years, 200 thieves and murderers, 280 invalids, attacked by blindness, idiocy or consumption, 90 prostitutes and 300 children who died prematurely. Out of 709 descendants, carefully studied, but few were honest. Of the men not more than twenty were skilled workmen, and ten of these learned their trade in prison.

---

CHRISTIAN science healers and faith curists are beginning to understand that the law must be respected, even if decency is ignored. A coroner's jury at Camden, N. J., on Saturday, December 18th, held two faith curists responsible for the death of a little two-year-old, Sarah Clare Kirby. She died last week under the treatment of her father, Frank Kirby, and a Philadelphia disciple, W. F. Randall. The prayers of these were unavailing, and after the authorities took charge and found another child ill from the same disease Kirby fought against the entrance of a physician to the house. Coroner Lippincott conducted the inquest. Kirby declared that at the birth of the last child he treated his wife by faith cure with success. The birth of the child was certified to by a physician who was called when it was all over, but Kirby would not allow the doctor to see his wife after that. He said he knew of other people being cured by Randall, who, he said, "charges a fee according to people's means." The child's life was insured, but Kirby did not know in whose favor.

The jury, after being out just long enough to frame a verdict, returned it as follows: "We find that Sarah Clare Kirby came to her death from diphtheria through the criminal negligence of her father, Frank Kirby, and we further find William F. Randall an accessory."

Coroner Lippincott committed Kirby in default of $500 bail. The next day he secured bondsmen and was released. The bondsmen were his father, Asa C. Kirby, also a faith curist, and William C. Kean. Randall has remained out of the state ever since the child died. He will be arrested in Philadelphia if he can be found. He will also be proceeded against for practising medicine contrary to the statute.

---

ANOTHER instance of the protective nature of the law against the ignorance of mankind is recorded in the *Western Medical Review* as follows: "Mrs. Baird, a christian science healer of Kansas City, was fined $50 on November 9th for failing to report to the board of health a case of diphtheria she was treating. The case was appealed. G. H. Kiney, the father of a little girl which had just died under Mrs. Baird's treatment, signed her appeal bond. The father and mother of a little girl who had died of diphtheria under christian science treatment were also arrested. They refuse to give the name of the christian (?) healer who attended their child."

---

SIR JAMES CRICHTON-BROWNE spoke at Selkirk, November 8, 1897, making the subject of his discourse Longevity and the brain, during which, according to the *London Lancet*, he dwelt on the dangers to health involved in indolence and disuse of the brain. The medical profession, he said, adapting itself to the needs of the times, had felt it incumbent upon it during the last decade to insist mainly on the evils of misuse of the brain, on the excessive strain not seldom imposed on it in these days in the fierce struggle of the race to be rich, and more especially on the over-pressure imposed on it in the name of education when in an immature state, but they were not less keenly alive to the correlative evils of the disuse of the brain.

Elderly persons who gave up business and professional men who laid aside their avocations without having other interests or pursuits to which to turn were in many cases plunged into despondency or hurried into premature dotage. He did not know any surer way of inducing premature mental decay than for a man of active habits to retire and do nothing when just past the zenith of life; and, on the other hand, he did not know any surer way of enjoying a green old age than to keep on working at something till the close. It had been said that one of the rewards of philosophy was length of days, and a striking list might be presented of men distinguished for their intellectual labors, which they had never laid aside, who had far exceeded the allotted span of human life. Galileo lived to seventy-eight, Newton to eighty-five, Franklin to eighty-five, Buffon to eighty, Faraday to seventy-six, and Brewster to eighty-four years. Sir James Crichton-Browne drew special attention to the great age generally attained by our judges.

---

In reporting the proceedings of the recent meeting of the Southern Surgical and Gynecological Association, the St. Louis *Globe-Democrat* prints the following interesting paragraph relating to the official stenographic reports of society meetings :

"One of the most interesting talkers of the meetings is Dr. William Whitford, of Chicago, but all his talking is done in the corridors before and after the sessions, as he is much too busy when the surgeons are in session to do anything but take the proceedings in shorthand. Dr. Whitford is the society's official stenographer. For twelve years his sole occupation has been the reporting of proceedings of medical societies. He probably occupies a field in which he has practically no competition, as fourteen societies engage his services almost constantly. He attended the college of medicine at Ann Arbor, Mich., in connection with the University of Michigan. Being a rapid shorthand writer, he was engaged during his college career to report medical lectures. This gave him a reputation which enabled him to spend his entire time at a calling in which the ordinary court reporter or shorthand writer would be lost. His greatest work is at the meetings of the American Medical Society. It has 9,000 members, and he is forced to employ eleven assistants. The longest discussion he ever 'took' was one lasting three hours on the subject of appendicitis, and 30,000 words were delivered. They were not the ordinary words which a shorthand man is used to taking, but words like staphylococcus, laparomyomectomy and cholecyst-enterost-omy came at the rate of 200 per minute—for the surgeons are fast talkers. Dr. Whitford was asked if he ever found a sentence which he could not 'take,' and was forced to admit that he had—it was before the American Association for the Advancement of Science, and the theme under discussion was the aurora borealis. The sentences which made Whitford stumble with his pencil, if his own statement is accepted, were : 'When the melofygistic temperature of the horizon is such as to calorcise the impurient indentation of the hemispheric analogy, the cohesion of the borax curbistus becomes surcharged with infinitesimals which are thereby deprived of their fissaral disquisitions. This effected, a rapid change is produced in the thorambumpter of the gyasticutis paleorum. This causes a convacular in the hexagonal antipathies. The clouds become a mass of deodorised spreula of cermocular light, which can only be seen when it is visible.' "

---

If it is true that more needy persons have applied to the poor department for aid at this season of the year than usual, why is there not a fine opportunity for the municipality to set these men to work upon the streets and thus make them self-supporting? We have heretofore pointed out not only the propriety but the economy of such a course. If the poor and needy are given employment, they are not only enabled thus to earn their own living, but occupation so afforded is a powerful factor in the prevention of disease. Main street from the harbor to the crossing of Chippewa street should be cleared of snow as soon as it falls. The wide space cleared at the crossings and in the center by the street cars ruins the sleighing, hence it were much better to clear the entire street in the section we mention.

